# Machine Learning Driven Fluidity and Rheological Properties Prediction of Fresh Cement-Based Materials

**DOI:** 10.3390/ma17225400

**Published:** 2024-11-05

**Authors:** Yi Liu, Zeyad M. A. Mohammed, Jialu Ma, Rui Xia, Dongdong Fan, Jie Tang, Qiang Yuan

**Affiliations:** 1 School of Civil Engineering, Central South University, Changsha 410075, China; liu_yi@csu.edu.cn (Y.L.); 224818004@csu.edu.cn (Z.M.A.M.); 234811013@csu.edu.cn (J.M.); 244812221@csu.edu.cn (R.X.); 2 National Engineering Research Center of High-Speed Railway Construction Technology, Changsha 410075, China; 3 Anhui Engineer Material Technology Co., Ltd. of CTCE Group, Hefei 230041, China; 13517400166@163.com (D.F.); 18978990472@163.com (J.T.)

**Keywords:** machine learning, workability, rheological property, feature importance analysis

## Abstract

Controlling workability during the design stage of cement-based material mix ratios is a highly time-consuming and labor-intensive task. Applying artificial intelligence (AI) methods to predict and optimize the workability of cement-based materials can significantly enhance the efficiency of mix design. In this study, experimental testing was conducted to create a dataset of 233 samples, including fluidity, dynamic yield stress, and plastic viscosity of cement-based materials. The proportions of cement, fly ash (FA), silica fume (SF), water, superplasticizer (SP), hydroxypropyl methylcellulose (HPMC), and sand were selected as inputs. Machine learning (ML) methods were employed to establish predictive models for these three early workability indicators. To improve prediction capability, optimized hybrid models, such as Particle Swarm Optimization (PSO)-based CatBoost and XGBoost, were adopted. Furthermore, the influence of individual input variables on each workability indicator of the cement-based material was examined using Shapley Additive Explanations (SHAP) and Partial Dependence Plot (PDP) analyses. This study provides a novel reference for achieving rapid and accurate control of cement-based material workability.

## 1. Introduction

The fluidity and rheological properties of cement-based materials serve as primary evaluation indicators for workability, closely associated with the processes of transportation, pumping, pouring, and molding in construction [1,2,3,4,5]. The regulation of early workability is a pivotal process in the mix design of cement-based materials, as it directly impacts their operability, quality during construction, and performance of the final product [6,7,8]. Exceptional workability ensures smooth flow and complete mold filling at construction sites, effectively preventing segregation, bleeding, and other undesirable issues in cement-based materials [9,10]. This guarantees material uniformity, structural integrity, as well as outstanding mechanical properties and durability after hardening [11,12,13]. Therefore, in the design of cement-based materials, excellent workability not only meets construction requirements but also ensures the quality and long-term performance of the final project [14].

In the design process of cement-based materials, the accurate estimation of mechanical properties can be achieved through the utilization of numerous empirical formulas [15,16,17]. Furthermore, different design standards impose specific requirements on cement, water, and aggregate contents, thereby simplifying the material design process for mechanical properties. Compared to the mechanical properties, the factors influencing early workability are more complex, making accurate estimation based on simplified assumptions challenging [18,19]. Therefore, regulating workability during material trial mixing becomes crucial and often consumes a significant amount of time in designing material mix proportions. The limitations of traditional design methods reduce the efficiency of material design. The introduction of a new design paradigm is crucial for improving the efficiency and precision of workability regulation [20,21,22].

The rheological properties describe the flow and deformation characteristics of fresh cement-based materials, which are closely linked to their fluidity, cohesiveness, and time-dependent behavior [23,24,25]. Under a constant shear rate, the dynamic yield stress represents the minimum shear stress required for the fresh cement-based material to begin steady flow. This refers to the minimum shear stress required for flow to begin at a given shear rate. Cement-based materials exhibiting an appropriate dynamic yield stress demonstrate improved flow stability and uniformity during the flow process, which is crucial for evaluating their flow capacity during the initial phases of construction [26,27]. The internal frictional resistance of fresh cement-based materials governs their deformation and flow behavior prior to and following the onset of flow. Additionally, plastic viscosity quantifies the additional shear stress required to maintain continuous material movement beyond the yield stress. A low plastic viscosity indicates that the material flows easily under external forces, while a high plastic viscosity indicates that considerable shear stress is needed to maintain a high shear rate during the flow process [28,29]. The dynamic yield stress characterizes the material’s resistance to the initiation of flow, while plastic viscosity quantifies its ability to maintain a flowing state once movement has started. In construction, cement-based materials are typically subjected to varying shear rate conditions, such as during vibration and flow operations. Precisely designing the rheological properties is essential for enhancing the stability of material flow under continuous construction conditions [30].

As the requirements for cement-based materials continue to evolve, the utilization of admixtures and supplementary cementitious materials has become increasingly widespread [31,32,33,34]. The incorporation of various components has presented greater challenges in controlling the workability of cement-based materials, increasing the complexity of evaluating their early-age performance [35,36]. The influence of superplasticizer (SP), viscosity-modifying agents, silica fume (SF), and fly ash (FA) on flowability and rheological properties is complex and nonlinear, with mutual interactions and synergistic effects contributing to the overall behavior of cement-based materials [37,38]. The predictive models for rheological properties and fluidity developed through traditional empirical methods show considerable limitations. With the increasing diversity of raw material components in cement-based materials, the challenge of rapidly and accurately designing mixes to optimize workability remains considerable [39,40,41,42].

The growing adoption of artificial intelligence (AI) methods, particularly machine learning (ML) and data-driven approaches, has enabled more accurate prediction and optimization of material mix proportions, thereby facilitating precise performance prediction and optimization [43,44,45,46]. ML has the capability to automatically extract patterns and relationships from datasets, enabling more effective data prediction and analysis. It has been extensively studied and applied in various fields, including material performance prediction, intelligent monitoring, and mix proportion optimization [47,48,49]. This data-driven design approach addresses the limitations of traditional empirical formulas, allowing for the modeling and prediction of complex material performance relationships. As a result, it offers more precise and reliable design solutions for challenging problems in the field of cement-based materials [50,51,52,53]. The application of ML and other advanced AI methods provides a reliable basis for predicting and rapidly designing the workability of fresh cement-based materials [54,55,56]. The utilization of reliable predictive models can significantly simplify the process of regulating material workability. Therefore, it is essential to validate the feasibility and reliability of ML methods in predicting the workability of cement-based materials, thereby further exploring their potential in supporting the design process. Moreover, the data-driven analysis approach can also provide deeper insights into the workability design of cement-based materials. It offers a more intuitive reference for optimizing material proportions.

This study established a comprehensive dataset through experimental testing, followed by the development of an ML-based prediction model for the fluidity, dynamic yield stress, and plastic viscosity of cement mortar. During model training, hyperparameters were optimized using an optimization algorithm to enhance model performance. Additionally, SHAP and PDP methods were employed to analyze the interpretability of the prediction model and explore the influence of cement-based material components on workability. This study aims to employ data-driven approaches to facilitate rapid and accurate prediction, as well as the efficient design and control of workability in cement-based materials.

## 2. Methodology

### 2.1. Experimental Methods

#### 2.1.1. Materials

Ordinary Portland cement (P.O. 42.5, supplied by Sinoma Cement Co., Ltd., Beijing, China), silica fume (SF, Sichuan Langtian Resource Comprehensive Utilization Co., Ltd., Chengdu, China), and class F fly ash (FA, Hunan Huadian Changde Powder Generation Co., Ltd., Changde, China) were used as cementitious materials in this paper. The chemical and mineral compositions, as shown in Table 1, were determined by X-ray fluorescence elemental analysis (XRF). River sand was used as aggregate, and a 2.36 mm sieve was employed to segregate larger particles to ensure the precision of the rheological test. The superplasticizer (SP, produced by Shanxi Feike New Materials Technology Co., Ltd., Yuncheng, China) is a polycarboxylate superplasticizer (powder), and hydroxypropyl methyl cellulose (HPMC, Shaanxi Hengxiang Chemical Technology Co., Ltd., Xi’an, China) was used as a viscosity-modifying agent.

#### 2.1.2. Fluidity Test

The fluidity of mortar was tested using the flow table test method, as specified by Chinese standard GB/T 2419-2005 [57]. Fresh mortar was poured into a cone mold and compacted thoroughly to ensure uniformity. After removing the mold, the flow table was operated for 25 cycles at a frequency of one cycle per second. Finally, the spread diameter (mm) was measured.

#### 2.1.3. Rheological Test

The rheological properties of fresh cement mortar were evaluated using a coaxial cylinder rotary rheometer (Rheolab QC, Anton Paar, Graz, Austria) equipped with a four-blade rotor (type ST22-4V-40). The rheological testing consisted of two phases: initially, a shear rate of 100 s−^1^ was applied for 1 min, followed by sequential shear rates of 100, 80, 60, 40, and 20 s−^1^. Each shear rate was maintained for 30 s, during which a total of 60 data points were collected. The average of the final 30 data points at each step was then used to model the rheological properties using the Bingham model.

### 2.2. Data Collection and Statistical Analysis

#### 2.2.1. Mix Proportion Data Generation

The programming language Python 3.9 was used to implement all experiments and analyses in this study. All the data used in this paper were collected from experimental test results obtained through the previously described methodologies. To enhance the quality of the dataset and ensure the generalizability of the prediction model, the tested mix proportion data were randomly generated within a constrained framework. The generated data represent the proportion of all components, expressed as percentages (%). The experimental data generation was constrained according to the following criteria:

1 Firstly, the sum of cementitious materials is randomly generated, with a minimum requirement that cement content should not be less than 70% of the total cementitious materials and SF content should not exceed 15% of the total cementitious materials.
2 Additionally, if the randomly generated values for both FA and SF are too low, a value of 0 is directly assigned to each.
3 The water content constitutes 25–65% of the total cementitious materials, while the sand content ranges from 1.5 to 4 times the total content of cementitious materials.
4 The addition of SP should fall within the range of 0.4–1.2% of the total cementing material, and the inclusion of HPMC should be considered within a range of 0.05–0.4%. Additionally, certain sample values may be randomly set to zero.
5 When the sand-to-cement ratio is excessively high, it is necessary to increase both the w/b and the dosage of SP to ensure accurate rheology testing results.
6 The obtained random data should be converted to sum up to 1.

Following these principles, a mix proportion dataset consisting of 240 samples was formed. Subsequently, employing the aforementioned testing methods enabled the determination of fluidity, dynamic yield stress, and plastic viscosity for each individual sample.

#### 2.2.2. Statistical Overview of the Data

**Figure 1:**
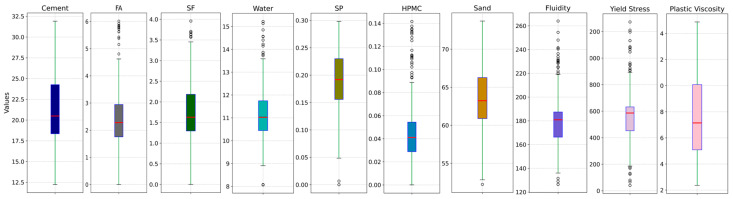
Boxplots of the original variables.

After establishing the dataset, outliers were eliminated to enhance the quality of the dataset. In the initial dataset, outliers may result from experimental errors or random fluctuations during data generation. These outliers can negatively affect model training, especially when employing nonlinear ML models like XGBoost and SVR, which are prone to overfitting on extreme data points and reduce generalization on the test set. Therefore, removing outliers helps the model to more accurately capture the overall data patterns, thereby mitigating the risk of overfitting to a few unrepresentative samples. To enhance the robustness of the trained model and improve its adaptability to real engineering data, the outliers of the input parameters shown in the boxplots (Figure 1) were retained. The outliers in the output parameters had a significant impact on model performance; therefore, those with substantial deviations were removed. Table 2 provides an overview of the distribution properties associated with both input and output variables within this dataset.

**Figure 2:**
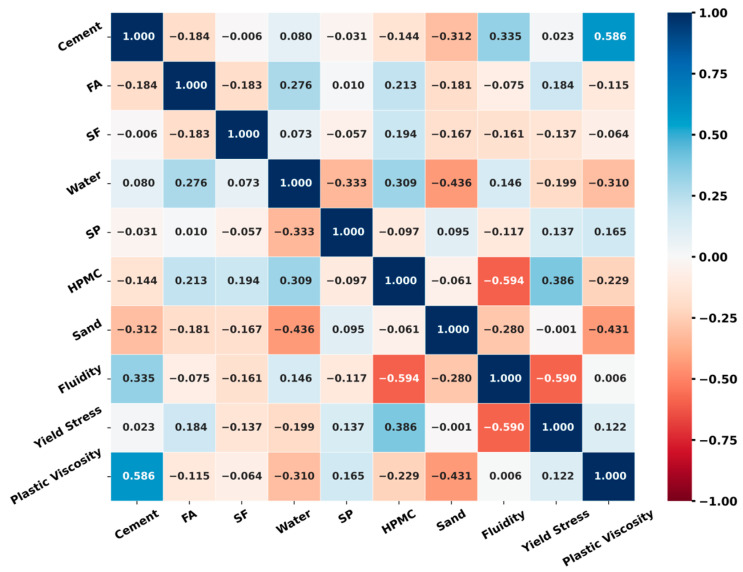
Pearson correlation coefficient of each factor.

**Figure 3:**
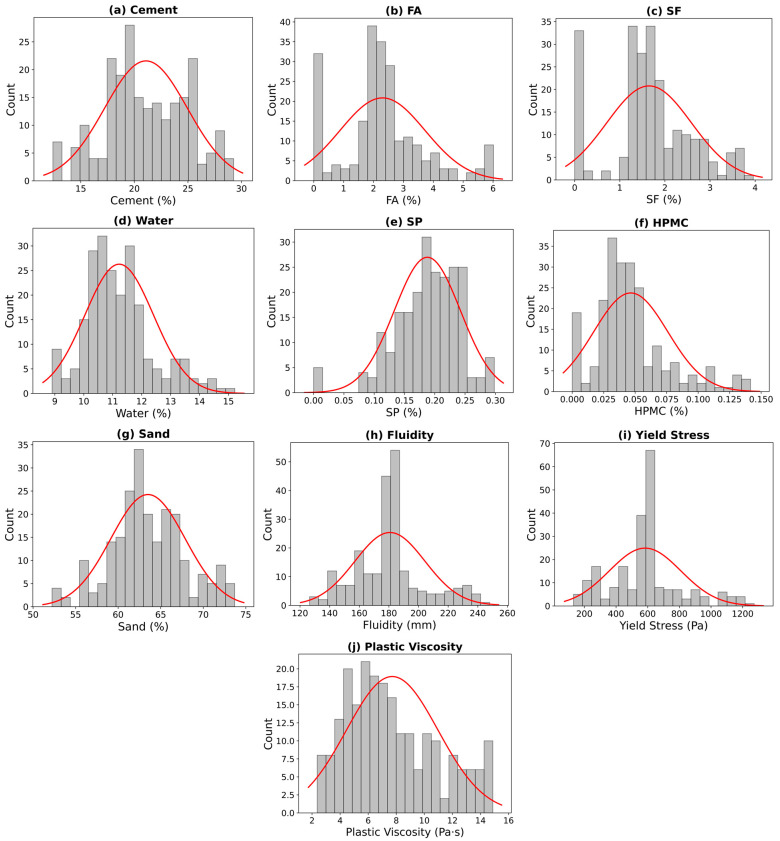
Statistical analysis of the variables.

The Pearson correlation coefficients of the variables are depicted in Figure 2. The Pearson correlation coefficients of the input variables are all below 0.6, indicating that the selected input variables will not produce multicollinearity problems. The statistical analysis of the variables is shown in Figure 3.

#### 2.2.3. Data Preprocessing

The significant discrepancy in the numeric values of different variables can substantially impact the performance of ML models. To mitigate this, it is often beneficial to standardize or normalize the input and output data to prevent overfitting caused by inconsistent numerical scales. The process of standardization involves transforming data into a standard normal distribution characterized by a mean of 0 and a variance of 1. By standardizing the data, dimensional differences can be eliminated, and the features can be ensured to exhibit similar distributions. On the other hand, normalization entails scaling the data to fit within a specific interval, typically ranging from 0 to 1, ensuring that all features fall within the same dimensional range. This approach proves particularly effective when there exists significant variation in feature values, as it facilitates faster convergence of gradient descent algorithms. The calculation methods for standardization and normalization are presented in Equations (1) and (2). In this paper, the data preprocessing of the fluidity and dynamic yield stress prediction model incorporates standardization to enhance prediction accuracy, while normalization is applied to the plastic viscosity prediction model.

(1) $$X_{\mathrm{standard}}=\frac{X-\mu }{\sigma }$$

(2) $$X_{\mathrm{normalized}}=\frac{X-X_{\min}}{X_{\max}-X_{\min}}$$

where *μ* is the mean value, *σ* is the standard deviation. X_max_ and X_min_ are the maximum and minimum values, respectively.

### 2.3. Modeling Methods

The data features in this study involve a relatively limited sample size and complex interactions among input variables. It is essential to select models capable of capturing high-dimensional, intricate relationships. During the model selection phase, a preliminary comparison of several widely used ML algorithms was conducted, with prediction accuracy and the potential for overfitting as the principal criteria for evaluation. As a result, SVR, XGBoost, and CatBoost were selected as the primary models for this study. A concise overview of the selected ML models is as follows.

#### 2.3.1. Support Vector Regression (SVR)

Support vector regression (SVR) is a regression algorithm that leverages the principles of support vector machines (SVM). Its fundamental concept involves identifying a high-dimensional regression function capable of minimizing errors, ensuring most data points are closely aligned with the model rather than being strictly fitted. Its advantage lies in processing nonlinear data, capturing complex data relationships by mapping data to high-dimensional spaces using kernel functions. SVR also introduces relaxation variables, which can find smoother solutions in noisy data. Therefore, it is suitable for small sample sizes and high-dimensional data scenarios. The objective function of SVR and the RBF kernel function used in this study are presented in Equations (3) and (4), respectively.

(3) $$\underset{w,b}{\min}\frac{1}{2}{\left\|w\right\|}^{2}+C\sum_{i=1}^{n}\left[\max\left(0,\left|y_{i}-\left(w^{T}\emptyset \left(x_{i}\right)+b\right)\right|-\varepsilon \right)\right]$$

(4) $$\emptyset \left(x_{i},x_{j}\right)=\exp\left(-\frac{{\left\|x_{i}-x_{j}\right\|}^{2}}{2\sigma^{2}}\right)$$

where *w* is weight vector, *b* is the bias term, ε is the error threshold, $\emptyset \left(x_{i},x_{j}\right)$ is the kernel function, and C is the regularization parameter.

#### 2.3.2. Extreme Gradient Boosting (XGBoost)

Extreme Gradient Boosting (XGBoost) is an exceptionally efficient ML algorithm based on the gradient boosting framework and is extensively employed for both classification and regression tasks. It mitigates model complexity through the application of L1 and L2 regularization techniques, reducing the likelihood of overfitting and enhancing the model’s generalization capabilities. The objective function and regularization term are shown in Equations (5) and (6). Furthermore, XGBoost can handle missing values in data without any prior preprocessing, making it one of the toughest algorithms for dealing with datasets containing several missing values and outliers.

(5) $$obj=\sum_{i=1}^{n}l\left(y_{i}-\overset{\wedge }{y_{i}}\right)+\sum_{t=1}^{T}\Omega \left(f_{t}\right)$$

(6) $$\Omega \left(f_{t}\right)=\gamma T+\frac{1}{2}\lambda \sum_{j=1}^{T}w_{j}^{2}$$

where $l\left(y_{i}-\overset{\wedge }{y_{i}}\right)$ is the loss function, $\Omega \left(f_{t}\right)$ is the regularization term, *T* is the number of leaf nodes in the tree, γ is the penalty parameter for leaf node complexity, $w_{j}$ is the weight of the j-th leaf node, and λ is the weight regularization coefficient.

#### 2.3.3. Categorical Boosting (CatBoost)

Categorical Boosting (CatBoost) is an ML algorithm based on gradient boosting over decision trees, specifically designed for effective regression tasks. It utilizes innovative techniques such as symmetric tree construction and ordered boosting to greatly enhance predictive accuracy and generalization. The objective functions of CatBoost and XGBoost in regression problems are fundamentally similar; however, the CatBoost algorithm incorporates certain regularization techniques not provided in XGBoost. Additionally, its sequential boosting mechanism is superior at eliminating overfitted models while opting for good generalization on smaller datasets. This is crucial for high-dimensional or limited-sample datasets where traditional models could struggle with either overfitting or poor generalization.

#### 2.3.4. Particle Swarm Optimization (PSO)

Particle Swarm Optimization (PSO) is a swarm intelligence-based optimization algorithm in which each particle represents a candidate solution. PSO simulates the movement of particles within the search space to find the optimal solution. These particles adjust their positions based on both their own experiences and the collective knowledge of the swarm, gradually converging toward the global optimum. In hyperparameter optimization, PSO serves as a global search algorithm capable of searching through a complex search space to find suitable hyperparameter combinations. The position of each particle represents a specific set of hyperparameters, which are evaluated based on a target function, such as model accuracy or loss. Particles update their velocity and position by considering both their locally discovered optimum and the globally identified optimum from the entire swarm, thereby approaching an optimal hyperparameter configuration.

#### 2.3.5. Shapley Additive Explanation (SHAP)

The Shapley Additive Explanation (SHAP) method is utilized to visually elucidate the contributions and impacts of each feature on predicted outcomes, thereby enhancing the interpretability of ML models. For each sample, SHAP generates a predicted value by quantifying the contribution of each feature to the prediction. This approach facilitates both global and local interpretations of model predictions by effectively decomposing complex models into individual feature contributions, providing an intuitive understanding of the influence each feature has on the predicted results.

#### 2.3.6. Partial Dependence Plot (PDP)

PDP considers all samples to determine the relationship between feature values and predicted outcomes. By varying a specific feature across different values, PDP effectively captures the dynamic patterns of model outputs, revealing the complex relationship between the feature and the target variable. The primary advantage of PDP is its ability to independently assess the impact of features on predictions, thereby highlighting the model’s sensitivity to specific features. Additionally, multidimensional PDPs enable the examination of interaction effects among multiple features.

## 3. Models Optimization and Evaluation

To establish efficient ML models, careful adjustment of hyperparameters is imperative, as these significantly influence the architecture and performance of the models. In order to ensure the validity of the trained model, the dataset is partitioned into two distinct subsets: the training set and the test set. The training set comprises 80% of the data used for model training and hyperparameter adjustment, while the remaining 20% is reserved for the independent test set to assess the generalization performance of the trained model as well as its accuracy. For rigorous evaluation and verification of predictive accuracy, it is essential to maintain strict independence between the test set and the training dataset, which will guarantee that the model’s performance is assessed on unseen data, providing an unbiased measure of its predictive capability.

### 3.1. Performance Evaluation

The prediction accuracy and generalization ability of ML models can be comprehensively demonstrated by simultaneously considering multiple evaluation indexes. The performance of ML models is further evaluated using common performance metrics such as the mean absolute error (MAE), the root mean square error (RMSE), the coefficient of determination (R^2^), and the mean absolute percentage error (MAPE). R^2^ offers an intuitive evaluation criterion for assessing the goodness of fit, representing how close the predicted values are to the true values. RMSE represents the deviation between the actual and predicted values, exhibiting heightened sensitivity towards significant discrepancies and effectively highlighting the impact of outliers on model performance. MAPE represents the mean of the absolute percentage error between the actual and predicted values, providing a measure of the average magnitude of prediction errors in terms of relative percentages. The corresponding formulas are given below.

(7) $$MAE=\frac{1}{n}\sum_{i=1}^{n}\left|{\hat{y}}_{i}-y_{i}\right|$$

(8) $$RMSE=\sqrt{\frac{1}{n}\sum_{i=1}^{n}{\left(y_{i}-{\hat{y}}_{i}\right)}^{2}}$$

(9) $$R^{2}=1-\frac{\sum_{i=1}^{n}{\left(y_{i}-{\hat{y}}_{i}\right)}^{2}}{\sum_{i=1}^{n}{\left(y_{i}-\bar{y}\right)}^{2}}$$

(10) $$MAPE=\frac{100\%}{n}\sum_{i=1}^{n}\frac{\left|y_{i}-{\hat{y}}_{i}\right|}{y_{i}}$$

where $y_{i}$ represents an actual data point from the dataset, $\bar{y}$ is the mean value of actual data, ${\hat{y}}_{i}$ represents the corresponding value estimated by the ML model, and n represents the total number of data points.

### 3.2. Hyperparameters Setting

**Figure 4:**
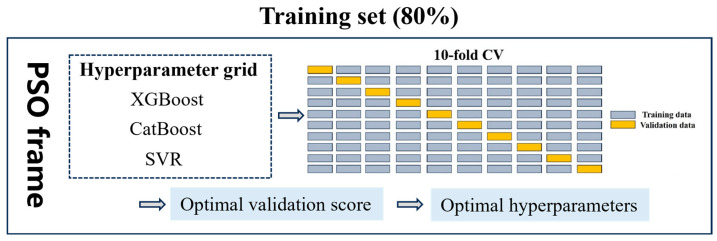
Hyperparameters optimization process.

This study presents two hyperparameter optimization methods, one of which combines random search and 10-fold cross-validation (CV). In this method, the range for each hyperparameter is predefined, and random values are sampled within this range. The model performance is evaluated using 10-fold CV for each combination of hyperparameters. The proposed optimization method is employed for fluidity prediction, yielding superior optimization outcomes. The other approach involves the optimization of both the algorithm and hyperparameters, coupled with 10-fold CV. In this method, each hyperparameter combination is predetermined within a specific range. Particles are initialized with different hyperparameter combinations, with each particle randomly assigned values within the predefined upper and lower bounds specified for hyperparameter optimization. The fitness function evaluates each particle by training the model once, with the objective of minimizing the RMSE through 10-fold CV on the training set. This approach is employed to predict two complex material properties, plastic viscosity and yield stress, with the goal of optimizing model performance. The process of adjusting hyperparameters in this study is illustrated in Figure 4.

For the hybrid algorithm on dynamic yield stress and plastic viscosity prediction, the maximum number of iterations of PSO is 100, in which the learning factors c1 and c2 are 1.5 and 1.7, respectively, and the particle swarm size is 50.

## 4. Results and Discussions

### 4.1. ML Predictive Performance Analysis

**Figure 5:**
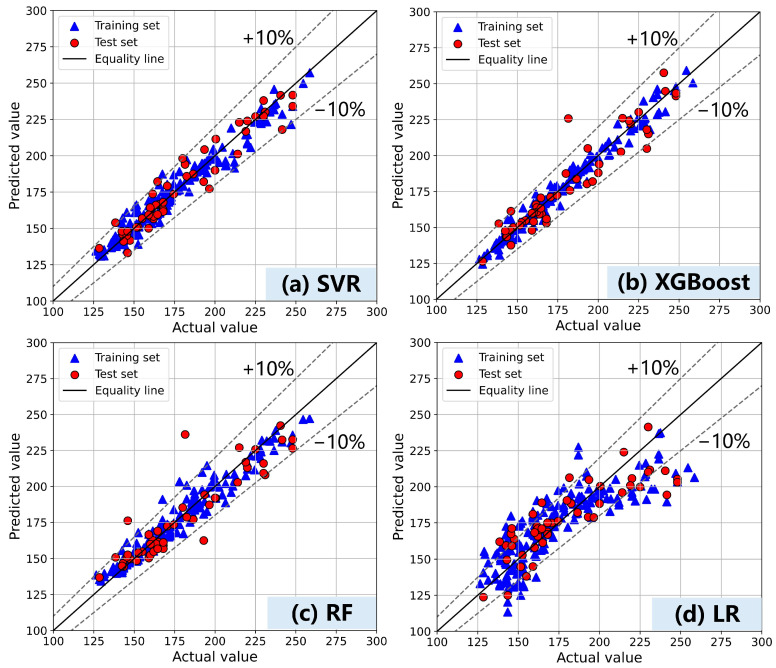
Performance of models on fluidity prediction (**a**) SVR, (**b**) XGBoost, (**c**) RF, (**d**) LR.

Figure 5 and Table 3 analyze the prediction results of fluidity by four ML models. Apart from LR, SVR, XGB, and RF also demonstrate commendable predictive performance in handling high-dimensional data. Notably, employing a straightforward random search coupled with 10-fold CV can yield satisfactory prediction results without necessitating complex hyperparameter optimization techniques. This indicates that, for data characterized by distinct global trends and localized nonlinear patterns, complex optimization algorithms may not be essential. By streamlining the optimization process, computational resources can be conserved while still attaining satisfactory accuracy, ensuring an efficient balance between performance and resource utilization.

SVR demonstrates optimal performance overall and is particularly suitable for processing data exhibiting local nonlinear patterns with clear global trends. Although RF performs well on the training set, its poor performance on the test set indicates overfitting. When the training dataset is limited in size, individual trees within the random forest model may struggle to capture the broader data patterns effectively. In contrast, the XGBoost algorithm, with its robust regularization mechanisms and enhanced gradient boosting capabilities, effectively mitigates errors and prevents overfitting, thereby exhibiting superior performance against RF on this dataset. However, SVR captures the most significant relationships, especially when confronted with data that exhibits local nonlinearities but with an easily observed global trend, which results in a more precise fit.

**Figure 6:**
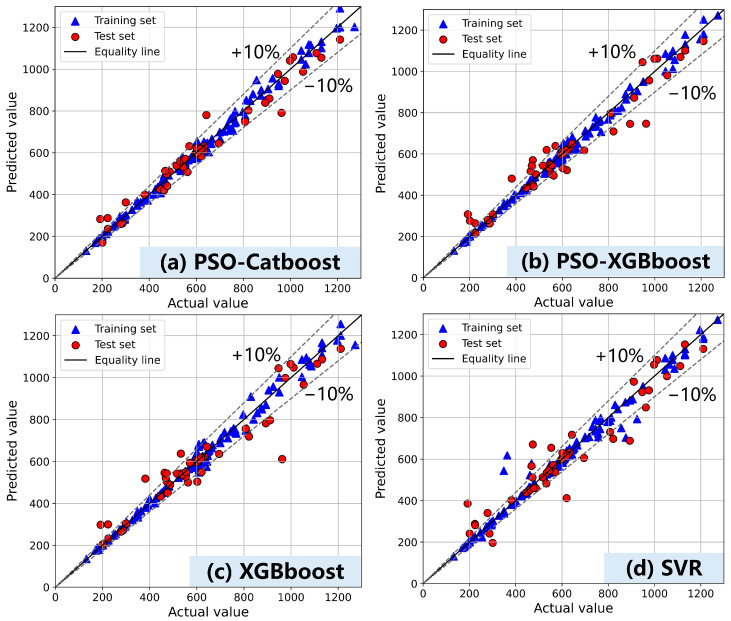
Performance of ML models on dynamic yield stress prediction (**a**) PSO-CatBoost, (**b**) PSO-XGBoost, (**c**) XGBoost, (**d**) PSO-SVR.

Figure 6 shows the prediction results of dynamic yield stress. The complexity of model selection and hyperparameter optimization for predicting dynamic yield stress is significantly increased due to the wide value span, varying numerical distributions among samples, and the complex interactions between features. These factors present considerable challenges to accurate prediction. In this context, CatBoost demonstrates excellent resistance against overfitting, with further improvement achieved through PSO optimization. The predicted values exhibit high accuracy, maintaining a 10% error margin and achieving an impressive R^2^ value of 0.94 on the test set. Additionally, the MAPE consistently remains low throughout the analysis. However, while XGBoost demonstrates excellent overall performance, it suffers from some overfitting and displays limited improvement when optimized using PSO. Meanwhile, the SVR model shows underfitting tendencies and has visible outliers in some of the training sets.

**Figure 7:**
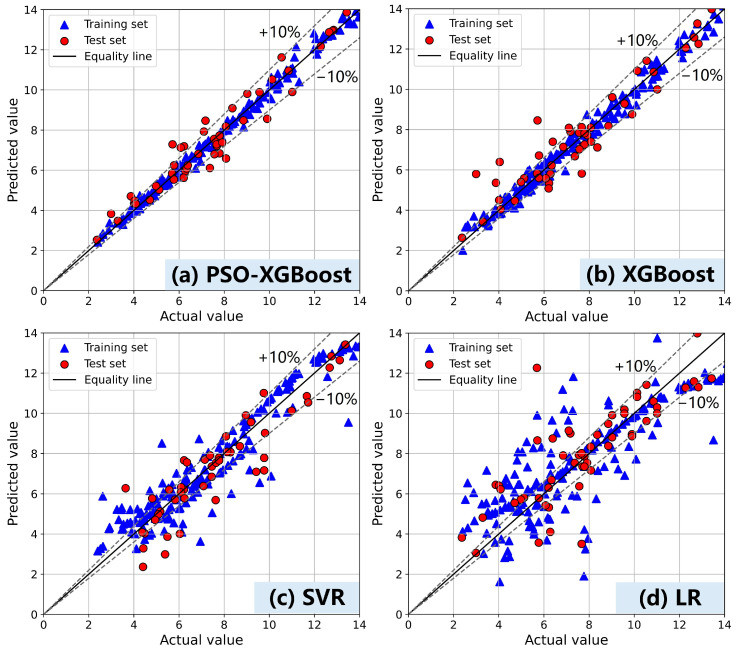
Performance of models on plastic viscosity prediction (**a**) PSO-XGBoost, (**b**) XGBoost, (**c**) SVR, (**d**) LR.

The prediction results for plastic viscosity are shown in Figure 7. Compared to the prediction of fluidity, the relationship between plastic viscosity and input features exhibits a higher level of complexity, resulting in highly dispersed prediction results from the LR model. The performance of SVR declines significantly in this case, as it is particularly well-suited for capturing local nonlinear relationships rather than addressing more complex global patterns. The PSO-optimized XGBoost model outperforms the XGBoost model with random search optimization, particularly in terms of its generalization ability. Although both models demonstrate similar R^2^ values on the training set (0.981 vs. 0.979), the PSO-XGBoost model exhibits notable advantages on the test set by effectively mitigating severe outliers and minimizing deviation. The PSO algorithm enables a more comprehensive exploration of the hyperparameter space, aiding in the identification of optimal hyperparameter combinations for high-dimensional data and complex feature interactions. This leads to a marked improvement in the generalization capacity of the ML model, as demonstrated by its superior handling of extreme values in the test set.

Furthermore, due to the complexity of the interrelationships within the data, predicting plastic viscosity is more difficult than predicting fluidity and dynamic yield stress. According to the Bingham model, the complexity of plastic viscosity arises from its dependence not only on the initial state of the material but also on structural evolution and particle redistribution during continuous flow. Plastic viscosity is highly sensitive to the specific details of particle distribution, size, and shape. In contrast, dynamic yield stress primarily reflects the force required to overcome the material’s initial state of rest. It is primarily influenced by the material’s macroscopic structural characteristics, making it less sensitive to minor fluctuations in mixing proportions [58,59]. These factors contribute to the relative ease of predicting dynamic yield stress. Additionally, determining plastic viscosity through experimentation is more challenging and highly sensitive to minor variations in experimental conditions, especially in systems with high auxiliary cementing materials or high-viscosity pastes, where significant experimental errors may occur. On the other hand, dynamic yield stress better represents the initial state of the material, leading to greater numerical stability. These factors make it more difficult to fine-tune hyperparameters during the development of a plastic viscosity prediction model. The optimization algorithms efficiently explore the hyperparameter space using heuristic methods and intelligent analysis of objective function feedback, adapting well to complex data characteristics and enhancing overall model performance.

The evaluation metrics for all models and the corresponding optimal hyperparameters are presented in Table 3 and Table 4.

### 4.2. SHAP Analysis

**Figure 8:**
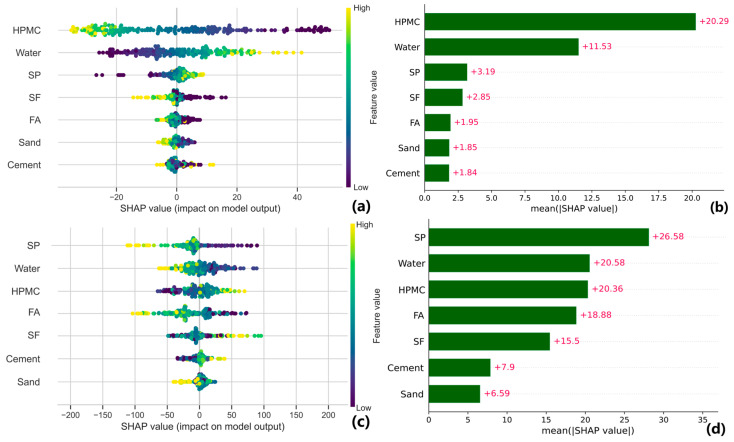

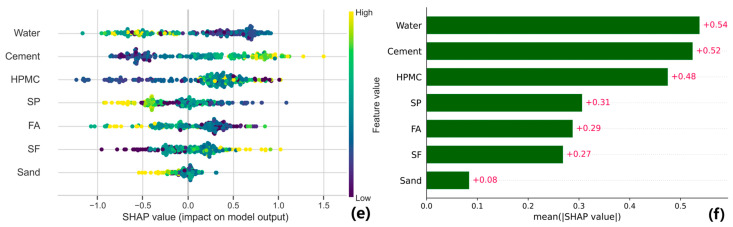
SHAP analysis on (**a**,**b**) fluidity, (**c**,**d**) dynamic yield stress, (**e**,**f**) plastic viscosity.

Figure 8 shows the SHAP analysis results for fluidity, dynamic yield stress, and plastic viscosity. The summary of SHAP analysis is the global interpretation of all features on the modeling result. The SHAP values exhibit a positive correlation with higher feature values (the color is yellow), displaying a roughly symmetrical distribution around the center value of zero.

The SHAP analysis of fluidity is shown in Figure 8a,b. The characteristic values in the summary plot exhibit a symmetrical distribution from high to low on both sides of the SHAP = 0 axis, indicating a monotonic influence of all components on fluidity. HPMC has the most significant impact, with a SHAP value of 20.29, suggesting that altering the proportion of the viscosity modifier greatly affects material fluidity, primarily in a negative manner. Water follows as the next important factor, with a SHAP value of 11.53. The influence of SP, SF, FA, sand, and cement content ratios on fluidity is comparatively less pronounced than that of HPMC and water. It should be noted that SHAP results are closely related to actual material composition. In the mix proportion of this paper, due to its smaller base value, the range of change in the content of SP exhibits a significant degree of variability. Therefore, a change in the proportion of SP used multiple times could potentially have an equivalent impact on mobility as a 50% increase in the proportion of water utilized. This is the reason why water exhibits a greater influence compared to SP. Additionally, owing to its finer particle size distribution relative to FA and cement, SF exhibits higher SHAP values, further supporting the rationality of this prediction model. It is worth noting that SHAP does not establish an absolute physicochemical relationship between composition and performance but rather elucidates the relative impact of one feature on the model output compared to other features.

SP, HPMC, and water also significantly impact the dynamic yield stress prediction model. The primary role of SP is to increase the proportion of free water, thereby effectively reducing dynamic yield stress. HPMC notably enhances the resistance to segregation and bleeding in cement-based materials. As HPMC content increases, the slurry viscosity rises, improving overall stability. At the same time, water content reduces interparticle interaction forces, resulting in lower yield stress. The symmetrical SHAP summary plot for each feature in the predictive model clearly shows that the effects on dynamic yield stress are relatively balanced, indicating an approximately linear relationship between each feature and the target variables.

As shown in Figure 8e,f, water exerts the most extensive influence on plastic viscosity, exhibiting the widest range of SHAP values and the highest value at 0.54, indicating a significant reduction effect on plastic viscosity. Cement follows closely with an average SHAP value of 0.52 for plastic viscosity. Unlike in the prediction models for fluidity and dynamic yield stress, cement plays a more prominent role in this context. The increase in both the number and concentration of cement particles directly impacts the internal friction and flow resistance of the slurry, amplifying the effect of cement content on plastic viscosity [60,61]. By forming a network structure in water, HPMC effectively increases slurry viscosity. Sand exhibits the smallest SHAP value, with an average of 0.08, implying its limited contribution to plastic viscosity.

It is worth noting that in cementitious components, the SHAP values corresponding to higher FA are negative. This phenomenon is also observed in dynamic yield stress, implying that, in this prediction model, an increase in FA content leads to a decrease in the plastic viscosity of cement-based material. Although FA, SF, and cement are all cementitious materials, they influence plastic viscosity through different mechanisms. Cement particles are relatively large and irregular with rough surfaces, so the physical contact and friction between cement particles intensify under these conditions, resulting in higher slurry plastic viscosity. Due to its ultra-fine particle size, high water absorption capacity, and strong chemical reactivity, SF exhibits a remarkable ability to significantly enhance the plastic viscosity of cement-based materials. FA usually has a spherical shape with a smooth surface, enabling it to effectively mitigate friction among slurry particles. Moreover, the larger particle size of FA results in a lower concentration of solid particles in the slurry after its addition, thereby reducing internal particle friction and ultimately decreasing dynamic yield stress [62,63].

### 4.3. PDP Analysis

**Figure 9:**
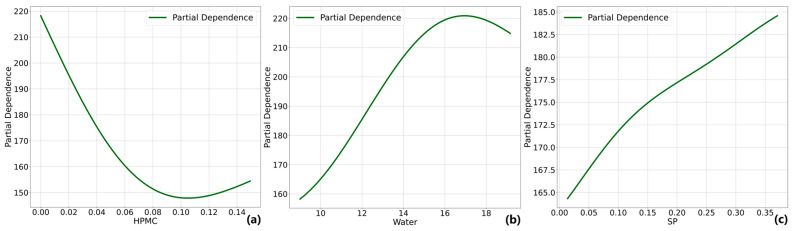
PDP analysis on fluidity (**a**) HPMC, (**b**) water, (**c**) SP.

**Figure 10:**
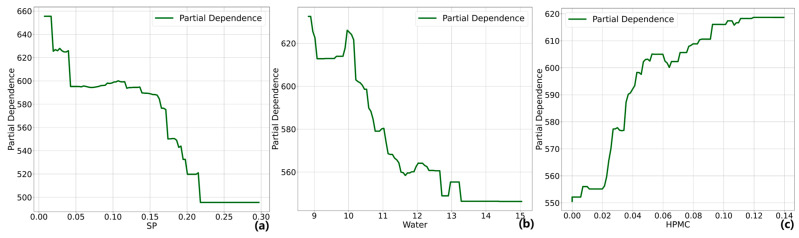
PDP analysis on dynamic yield stress (**a**) SP, (**b**) water, (**c**) HPMC.

**Figure 11:**
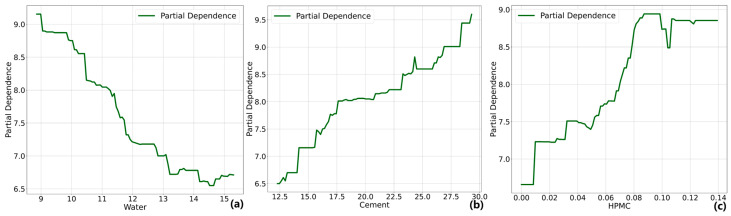
PDP analysis on plastic viscosity (**a**) water, (**b**) cement, (**c**) HPMC.

The influence of each design variable on the output variable can be explained by a Partial Dependence Plot. Figure 9, Figure 10 and Figure 11 analyze the influence of the major design variables on fluidity, dynamic yield stress, and plastic viscosity. In these figures, the horizontal axis represents the values of the design variables, while the vertical axis represents the variation in each workability. Similar to SHAP, PDP does not establish a causal relationship between components and performance but rather quantifies the relative impact on the predicted values of the model.

The role of HPMC, water, and SP in the prediction model of fluidity has been comprehensively analyzed using SHAP. As shown in Figure 9, the PDP analysis reveals that the influence of HPMC and water on fluidity is significantly associated with the range and proportion of their content. Specifically, when fluidity exceeds 150 mm, HPMC has a significant impact on reducing flow, but this effect diminishes when the flow is below 150 mm. Increasing the amount of HPMC from 0 to 0.08% leads to an average decrease in flow by 70 mm. On the other hand, for material flows lower than 220 mm, water plays a significant role in improving fluidity but loses its significance beyond this value. Increasing water content from 9% to 16% leads to an average fluidity increase of 60 mm. However, the relationship between water content and fluidity is not strictly linear. After a critical value (around 17%), further increases in water content do not substantially improve fluidity due to saturation effects on particle lubrication. When optimizing the material mix proportion, exceeding the water content threshold will not achieve the desired high fluidity and may instead lead to issues such as segregation or a reduction in strength. These observed findings are consistent with experimental results [60,64], which have shown that, after a certain threshold, increasing water content no longer improves fluidity but instead leads to segregation. In contrast, while the effect of SP does not diminish within its range, its overall average influence remains small compared to HPMC and water. Notably, the effect of SP closely correlates with water content such that it will be partially considered as increased water content within the prediction model that includes the effect of SP. So, the potential effect of SP might be underestimated.

The PDP results for dynamic yield stress are illustrated in Figure 10. The increase in SP and water content significantly reduces the yield stress of the cement-based material by dispersing the cement particles, diminishing the flocculation structure, and augmenting the free water content in the slurry. This effect reduces the shear stress required for slurry flow initiation. When SP exceeds 0.21% and water surpasses 13.3%, a plateau is observed in the PDP curve, indicating potential segregation or bleeding phenomena where further reduction in yield stress around the rheometer rotor becomes insignificant. This suggests that the excess of SP and water can compromise the structural integrity of the slurry, hindering effective interaction and bonding between particles [65,66]. The influence of HPMC on yield stress is more pronounced within lower content levels.

In Figure 11, as the feature value of water increased, the PDP plot shows an almost consistent decline with a broad range of influence. However, with further increases in water content, the curve begins to plateau or even exhibit a slight upward trend, similar to the patterns observed in dynamic yield stress. This suggests the occurrence of material segregation and bleeding, indicating an unstable slurry structure. It is worth noting that the flat region observed in the PDP plot of dynamic yield stress and plastic viscosity approximately corresponds to the declining section in the fluidity curve. In rheological models, the fluidity of cement-based materials is typically characterized by these two parameters, and their combined effect determines the performance of fluidity. Once the water content reaches a critical point, the internal friction resistance of the material decreases to its minimum, making further reductions in yield stress and plastic viscosity insignificant. Consequently, the PDP plot for fluidity flattens or even begins to decline. The plastic viscosity of the paste gradually increases with the rise in cement content, and this increase becomes more pronounced at higher content. The effect of HPMC on plastic viscosity typically exhibits a non-linear relationship. As the content of HPMC increases, plastic viscosity experiences a rapid rise. After a higher content level, the increase in viscosity slows down, indicating a saturation of the thickening effect of HPMC.

## 5. Conclusions

This study conducted experimental tests to construct a comprehensive dataset encompassing the fluidity and rheological properties of cement-based materials. Through a framework utilizing random search and optimization algorithms, accurate machine learning prediction models were developed, enabling effective predictions of fluidity, dynamic yield stress, and plastic viscosity in cement-based materials. Furthermore, SHAP and PDP were used to reveal how variables specifically affected the fluidity, dynamic yield stress, and plastic viscosity of cement mortar. The resulting observations are as follows:

(1) The fluidity prediction model established in this paper accurately predicts fluidity within the range of 130–250 mm. The data complexity associated with the composition and fluidity of cement-based materials is low, enabling the SVR model to achieve precise predictions with high precision and strong generalization ability. Furthermore, both SHAP and PDP analyses demonstrate a relatively direct relationship between material components and fluidity, indicating weak interaction among these features.
(2) The dynamic yield stress prediction model established in this study demonstrates accurate prediction within the range of 200–1200 Pa, while the CatBoost model optimized by PSO exhibits superior performance. Under conditions of high SP and water content, the PDP curves for dynamic yield stress and plastic viscosity exhibit a flattening trend, which aligns with the behavior observed in the fluidity curve. This pattern suggests potential issues such as segregation and bleeding in the material.
(3) The PSO-optimized XGBoost model significantly outperforms the models optimized using random and grid search methods in predicting plastic viscosity, demonstrating the effectiveness of optimization algorithms for hyperparameter tuning in more complex data relationship prediction models. Additionally, the SHAP analysis revealed that SF, FA, and cement have different effects on dynamic yield stress and plastic viscosity prediction, which is closely linked to the physicochemical properties of the cementitious material particles.

## Figures and Tables

**Table 1:** Chemical compositions of cementitious materials (%).

	SiO_2_	Al_2_O_3_	Fe_2_O_3_	CaO	MgO	SO_3_	Other Minor Oxides	LOI
OPC	53.2	26.3	3.5	6.2	2.1	3.3	3.2	2.2
FA	48.4	29.4	7.8	4.7	0.6	0.7	3.3	5.1
SF	84.3	0.8	2.8	0.8	2.4	1.1	4.6	3.2

**Table 2:** Description of dataset used in this study.

Classification	Variable	Unit	Parameter Type	Data (233)			
				Min	Max	Ave	Med
Mixture Proportions	Cement	%	Input	12.23	31.91	21.08	20.59
	FA	%	Input	0.00	6.01	2.28	2.28
	SF	%	Input	0.00	3.96	1.62	1.64
	Water	%	Input	8.07	15.22	11.24	11.08
	SP	%	Input	0.00	0.30	0.19	0.19
	HPMC	%	Input	0.00	0.14	0.05	0.04
	Sand	%	Input	52.23	73.70	63.56	63.27
Workability	Fluidity	mm	Output	126.50	258.00	180.23	180.80
	Yield stress	Pa	Output	131.60	1272.50	579.92	589.82
	Plastic viscosity	Pa·s	Output	2.37	14.88	7.70	7.10

**Table 3:** Performance comparison of ML models.

	R^2^		RMSE		MAE		MAPE	
	Train	Test	Train	Test	Train	Test	Train	Test
Fluidity								
SVR	0.944	0.927	7.998	8.349	4.951	6.208	0.029	0.036
XGB	0.978	0.875	7.396	10.905	3.927	7.070	0.023	0.041
RF	0.939	0.796	6.814	13.179	4.870	8.615	0.027	0.048
LR	0.547	0.324	16.744	18.352	13.015	14.459	0.075	0.079
Dynamic yield stress								
PSO-CatBoost	0.984	0.941	24.019	45.567	12.181	36.539	0.021	0.063
PSO-XGBoost	0.989	0.895	23.274	50.769	11.022	41.758	0.019	0.072
XGBoost	0.985	0.844	26.732	76.309	14.501	46.848	0.025	0.084
PSO-SVR	0.931	0.853	31.744	77.149	19.141	60.321	0.033	0.104
Plastic viscosity								
PSO-XGB	0.981	0.927	0.163	0.836	0.123	0.571	0.020	0.076
XGB	0.979	0.875	0.321	1.030	0.232	0.717	0.038	0.104
SVR	0.880	0.838	1.059	1.204	0.805	0.878	0.122	0.130
LR	0.519	0.505	1.871	2.138	1.412	1.504	0.223	0.247

**Table 4:** Optimal hyperparameters of ML models for modeling fluidity, dynamic yield stress, and plastic viscosity.

Models	Hyperparameters
Fluidity	
SVR	C = 24.43, cache_size = 700, epsilon = 0.40, kernel = ‘rbf’, gamma = 0.08
XGB	n_estimators = 100, learning_rate = 0.10, gamma = 0.09, max_depth = 7, reg_alpha = 0.09, reg_lambda = 0.76, subsample = 0.6
RF	n_estimators = 58, min_samples_leaf = 2, min_samples_split = 5, max_depth = 9
LR	
Dynamic yield stress	
PSO-CatBoost	iterations = 687, border_count = 115, subsample = 0.403, learning_rate = 0.102, depth = 5, l2_leaf_reg = 1, rsm = 0.99
PSO-XGBoost	learning_rate = 0.101, n_estimators = 179, subsample = 0.648, reg_alpha = 0.578, reg_lambda = 0.589, max_depth = 7, gamma = 0.889
XGBoost	learning_rate = 0.148, n_estimators = 115, subsample = 0.62, reg_alpha = 0.22, reg_lambda = 0.46, max_depth = 5, gamma = 0.3
PSO-SVR	C = 56.17, epsilon = 0.330, gamma = 0.101, cache_size = 100, kernel = ‘rbf’
Plastic viscosity	
PSO-XGB	learning_rate = 0.121, n_estimators = 157, subsample = 0.656, reg_alpha = 0.552, reg_lambda = 0.893, max_depth = 4
XGB	learning_rate = 0.14, n_estimators = 115, subsample = 0.3, reg_alpha = 0.09, reg_lambda = 0.5, max_depth = 3, gamma = 0.3
SVR	C = 60.03, cache_size = 800, epsilon = 0.47, kernel = ‘rbf’, gamma = 0.418
LR	

## Data Availability

The original contributions presented in the study are included in the article, further inquiries can be directed to the corresponding author.
